# Early or recent trauma in treatment-resistant depression: a systematic review

**DOI:** 10.1017/S1092852925000185

**Published:** 2025-03-12

**Authors:** Sara Fantasia, Lorenzo Conti, Debora Andreoli, Andrea Bordacchini, Berenice Rimoldi, Valerio Dell’Oste, Virginia Pedrinelli, Claudia Carmassi

**Affiliations:** 1 Department of Clinical and Experimental Medicine, University of Pisa, Pisa, Italy; 2 Department of Mental Health and Addiction, Azienda USL Toscana Nord-Ovest, Lucca, Italy; 3 Department of Biotechnology- Chemistry and Pharmacy, University of Siena, Siena, Italy; 4 Department of Mental Health and Addiction, Azienda USL Toscana Nord-Ovest, Massa, Italy

**Keywords:** depressive disorder, treatment-resistant depression, trauma, childhood trauma, PTSD, stress, resistance

## Abstract

**Background:**

Increasing attention has been recently devoted to treatment-resistant depression (TRD); however, its clinical characteristics, potential risk factors, and course are still debated. Most recently, childhood trauma exposure has been correlated to TRD, but systematic investigation on the role of lifetime trauma is still lacking. The aim of this paper was to revise current evidence on early and recent trauma exposure in TRD.

**Methods:**

A systematic search was conducted from the 1st of June to the 20th of February 2024 in accordance with the PRISMA 2020 guidelines and using the electronic databases PubMed, Web of Science, and Embase.

**Results:**

The primary database search produced a total of 1998 record, and finally, the search yielded a total of 22 publications, including 18 clinical studies, 3 case reports, and 1 case series, all from the period 2014 to 2024.

**Limitations:**

Limitations include a small sample size of some studies and the lack of homogeneity in the definition of TRD. Furthermore, we only considered articles in English, we excluded preprints or abstracts, and we included case reports.

**Conclusions:**

This review highlights the role of early and recent trauma in TRD, even in the absence of a full-blown post-traumatic stress disorder (PTSD), highlighting the need for a thorough assessment of trauma in patients with TRD and of its role as a therapeutic target.

## 1. Introduction

Increasing attention has been recently devoted to treatment-resistant depression (TRD), suggesting it may affect from 20 to 40% of patients with major depression (MD), representing a very important clinical challenge in psychiatry. Individuals with TRD, in fact, tend to present a more severe and prolonged course of illness associated with a high risk for suicidal behavior, psychiatric and medical comorbidities, and greater social impairment.^1^ ^-^^3^ Nevertheless, TRD clinical characteristics, potential risk factors, and course are still debated.^1^ There are currently multiple different definitions of TRD, and this hinders a precise estimate of its prevalence, the identification of risk factors, and the optimization of effective interventions. Moreover, the missing of a consensus definition limits the interpretability and generalizability of the results of clinical studies due to the heterogeneity of populations enrolled.^1^ The most used definition of TRD is the one adopted by the US Food and Drug Administration (FDA) and the European Medicines Agency (EMA), and it’s the failure to respond to at least 2 appropriately prescribed antidepressant medications.^4^ ^,^^5^ Other definitions of the TRD include those of the Thase and Rush staging model, the Maudsley Staging Model (MSM), the European Group for the Study of Resistant Depression (GSRD), the Dutch Measure for quantification of TRD model (DM-TRD), and the Massachusetts General Hospital Staging Model (MGH-S).^1^ ^,^^6^ In the Thase and Rush model, patients are staged according to the number of classes of antidepressants that have failed to provide a response, with treatment resistance moving from more frequently used antidepressants to less frequently used agents.^7^ The MSM model uses the failure of the first antidepressant treatment to denote the treatment-resistance; additional focus is on both augmentation and ECT. The MSM includes additional clinical information on disease duration and severity to be added to the TRD level.^8^ The GSRD model distinguishes between non-response and resistance. The latter is applied after two or more adequate trials of different classes of antidepressants and is divided into five different levels of strength depending on the overall duration of the trials.^1^ ^,^^9^ The DM-TRD model considers many variables, adds functional impairment, anxiety symptoms, personality disorder, psychosocial stressors, different categories of augmentation/combination regimens, use of psychotherapy, and intensified treatment. This model is the most comprehensive in terms of variables included.^10^ The MGH-S separately considers dosage optimization and prolonged duration of treatment, as well as minimum operationalized dosage and duration; it does not provide an implicit hierarchy of antidepressant classes or an implicit preference for switching from one class to another over an internal class.^11^

Some authors have also pointed out that the concept of TRD has limitations, including the fact that the term evokes the idea of an inevitable negative outcome and implies an acute illness model with an exclusively biomedical approach to treatment, where resistance may be due to pharmacokinetic or pharmacodynamic mechanisms^12^ ^,^^13^. For this reason, an alternative concept has been proposed: “difficult-to-treat depression” (DTD), defined as “*depression that continues to cause significant burden despite usual treatment efforts.*” According to this definition, the “usual therapeutic efforts” are also dependent on the medical setting and environment and depend on local therapeutic practises; for the “burden” of illness, psychosocial functioning, and quality of life are also taken into account. In general, at least two treatment attempts should be considered, but in some cases, DTD may be suspected after only one treatment (eg, if the patient has many comorbidities or is receiving polypharmacotherapy).^14^

Although related to and partially overlapping with the concept of TRD, DTD recognizes more fully the complexity of managing depression and takes into account social and environmental factors that may stand in the way of recovery, leading to a more personalized approach that goes beyond standard treatments and involves shared responsibility between the clinician and the patient.^14^ Although doubts have also been expressed about this definition (the negative meaning of the word “difficult,” the risk of viewing the patient as difficult, and the risk of implicit blame), DTD is generally perceived as more open and collaborative^14^ and, most importantly, suggests that non-response to treatment in depression may be related not only to biological resistance but also to diagnostic inaccuracies, psychosocial variables, childhood maltreatment or trauma, job dissatisfaction, and physical and psychological comorbidities.^13^ ^,^^15^ This complexity confirmed by TRD literature suggesting the existence of several risk factors for the development of resistance such as a depressive episode in the context of bipolar disorder or with bipolarity features (eg, family history, subthreshold hypomanic episodes, hyperthymic temperament, and mixed features), low hedonic tone, attention-deficit hyperactivity disorder, anxiety comorbidity, psychotic features, a higher number of lifetime depressive episodes, partial remission, number of previous antidepressant trials, number of previous augmentation agents, previously failed psychotherapy, previous failed electroconvulsive therapy (ECT), the long duration of illness, symptom severity of the current episode, greater number of hospitalizations, prevalence of comorbidities (psychiatric and medical), comorbid personality disorder, and comorbid substance use.^16^ ^-^^22^ Most recent evidence suggests that psychosocial stressors and trauma are correlated with MD and mood disorders, as well as their more severe course of illness,^19^ ^-^^25^ so, there is now evidence indicating that the assessment of lifetime trauma exposure may be crucial to fully characterize patients with a major diagnosis of MD and should be part of accurate clinical assessment.^26^

Particular attention has been recently devoted to the role of childhood trauma exposure in the development and course of depression in adulthood. In particular, the association with earlier onset, higher symptom severity, number of comorbidities and relapses, suicidal behavior, and specific subtypes, such as those with psychotic and atypical features, has been highlighted.^2^ ^,^^27^ Childhood trauma also appears to affect treatment response, but literature data are inconclusive. Recently, the Childhood Trauma Meta-Analysis Study Group^28^ confirmed a high frequency of childhood trauma in depressed patients (62%). Interestingly, symptom improvement and dropout rates were similar to those in patients with depression without childhood trauma. This result contrasts with what has been reported in other work;^29^ the increased severity of symptoms after treatment seems to confirm that individuals with a history of child maltreatment are less likely to meet remission criteria than patients without a history of child maltreatment.^30^

Despite these premises, to our current knowledge, the role of trauma as a factor associated with TRD and its course has not been systematically investigated. Therefore, the aim of this review is to summarize the current knowledge on the relationship between trauma and TRD to improve the understanding of the disease.

## 2. Methods

### 2.1. Literature search

A systematic search was conducted from 1^st^ of June 2023 to 20^th^ of February 2024 in accordance with the PRISMA 2020 guidelines^31^ and using the electronic databases PubMed, Web of Science, and Embase. A combination of controlled vocabulary terms and free text terms, without filters, restrictions, or limits, was used to identify all potentially eligible records: in PubMed (((“Depressive Disorder, Treatment-Resistant”[MeSH Terms]) OR (“Treatment-Resistant depression”[All Fields]) OR (“treatment-refractory depression”[All Fields])) AND ((“Psychological trauma”[MeSH Terms]) OR (“Adverse Childhood Experiences”[Mesh]) OR (“early-life trauma”[Text Word]) OR (“trauma”[All Fields]) OR (“child abuse”[MeSH Terms]) OR (“child abuse”[All Fields]) OR (“neglect”[All Fields]) OR (“adversity”[All Fields]) OR (“stress”[All Fields]) OR (“stress event”[All Fields]))); in Web of Sciences ((ALL=(“Depressive Disorder, Treatment-Resistant”)) OR (ALL=(“Treatment-Resistant depression”))) AND ((ALL=(“Psychological trauma”)) OR (ALL=(Childhood trauma)) OR (ALL=(“early-life trauma”)) OR (ALL=(“trauma”)) OR (ALL=(“child abuse”)) OR (ALL=(“neglect”)) OR (ALL=(“adversity”)) OR (ALL=(“stress”)) OR (ALL=(“stress event”))); and in Embase (“treatment resistant depression”/exp OR “treatment-resistant depression” OR “treatment refractory depression”/exp OR “treatment refractory depression”) AND (“psychotrauma”/exp OR psychotrauma OR “early-life trauma” OR ((“early life”/exp OR “early life”) AND (“trauma”/exp OR trauma)) OR “child abuse”/exp OR “child abuse” OR “sexual abuse”/exp OR “sexual abuse” OR “neglect”/exp OR neglect OR “adverse event”/exp OR “adverse event” OR “physiological stress”/exp OR “physiological stress” OR “stress event” OR ((“stress”/exp OR stress) AND event)) .All studies from 1st January 1981 to 30th January 2024 were included in the database search.

### 2.2. Eligibility criteria

The criteria for inclusion of studies in this review were as follows:

1. Human studies
2. Study that used a validated scale to assess trauma and depression/resistant depression
3. Articles available in English

Because we aimed at investigating the relationship between trauma and TRD in patients, studies that examined this in animal models were excluded. Furthermore, preprints and publications in the form of abstracts, reviews, and editorials were also excluded. All authors agreed to include case reports.

### 2.3. Screening and selection process

**Figure 1.**
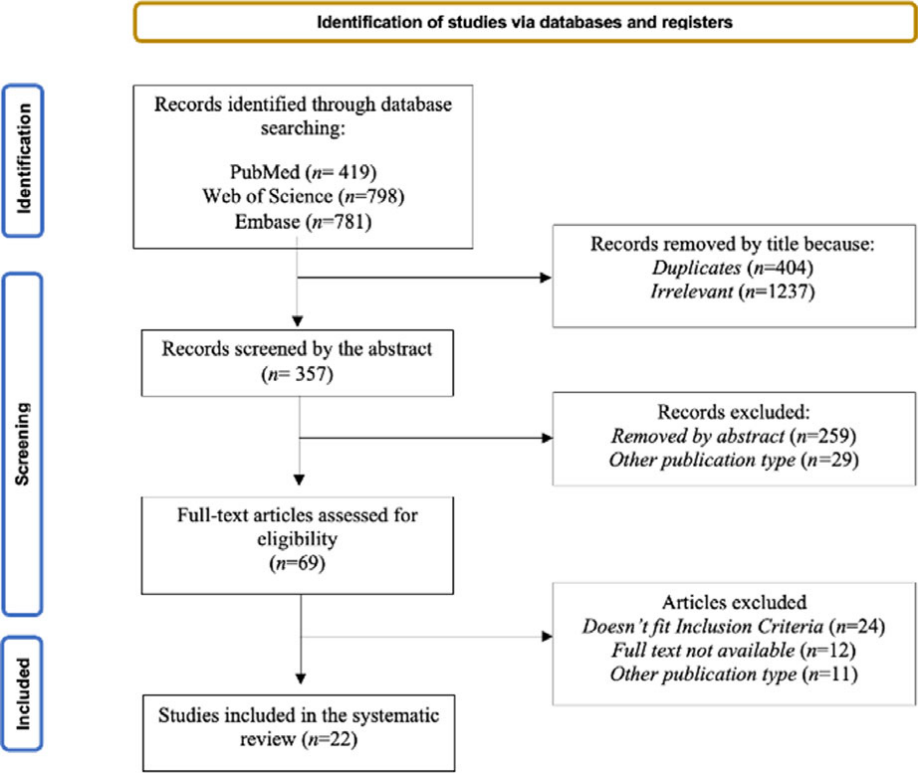
Flow chart.

Two independent reviewers (L.C. and D.A.) screened papers for inclusion. The primary database search produced a total of 1998 records. After that, 1641 articles were removed after titles because they were duplicates (N=404) or not relevant (N=1237), and 311 were removed after the abstract because they were not pertinent (N=259), full texts are not available or not in English (N=12), or because they were other publication types (N=40). After a full-text reading, another 24 articles were excluded because they didn’t fit the eligibility criteria. Finally, a total of 22 articles were included in the present review. All 2 reviewers completed the process independently. We assessed the reference lists of selected papers for other eligible studies, and any disagreement on included papers was resolved by discussion. Any disagreements were discussed and resolved by a third author, C.C. Decisions for inclusion or exclusion are summarized in a flowchart according to PRISMA 2020 recommendations.^31^ The study selection process is outlined in a flowchart (Figure 1).

### 2.4. Quality assessment

The quality of case reports included was assessed by a standardized tool adapted from Murad et al.^32^ Furthermore, we used the Quality Assessment Tool for Observational Cohort and Cross-Sectional Studies (QATOCCSS)^33^ to assess the quality of the other type of study. Each study was scored as either “good,” “fair,” or “poor” (see Table 1). The quality assessment was performed by 2 independent reviewers (L.C. and S.F.), and a third reviewer (C.C.) cross-checked the quality assessment result. Disagreements were discussed and resolved with the research team. The degree of agreement between the independent authors was good.

**Table 1.** Clinical Studies Included

Study	Year	Country	Quality rating	Type of study	Population	Sample		Mean age		TRD		Trauma		Type of intervention	Main finding
						Total TRD sample	Trauma	Total TRD sample	Trauma	Assessment	Previous treatment	Assessment	Type of trauma		
Tunnard et al.^2^	2014	UK	Good	Cross-sectional study	TRD patients	137	85 (62%)	47.9	*Not available*	SCID, MINI, BDI, HAM-D	At least one adequate antidepressant trial: a range of 1–19 and a median of 5 (IQR1/43–8). Implemented psychiatric medication trials: a median of 12 (IQR1/47–17) 85% h mood stabilizers; 80% an APS; 42% one or more other augmentation strategy; and 62% anxiolytic/hypnotic medication; 2/3 ECT therapy	ETI, CECA	Early childhood adversity (most frequent traumatic events, 35%)	-	Childhood adversity was common in TRD patients and was associated with poor clinical course, psychosis, and suicide attempts
Stevenson et al.^41^	2015	Australia	Fair	Observational study	TRD patients	44	44 (100%)	aged between 18 and 55 ys of age (*mean age not available)*		BDI, HAM-D GAF	At least three adequate trial of antidepressants as well as other classes of drugs (mood stabilizers, APS, BDZ), ECT, and psychotherapies (CBT, DBT, or supportive psychotherapy)	CTQ	early childhood trauma	CM of psychodynamic psychotherapy	High prevalence rates of early childhood trauma and personality disorders were found among patients with TRD. A trauma-informed therapy as in the CM along with pharmacotherapy resulted in symptomatic and functional improvement. Trauma was not mentioned in previous contacts, but was noted on assessment
Albott et al.^58^	2018	USA	Fair	Open-label study	Veterans with TRD and PTSD	15		52.1		SCID, MADRS, ATHF	At least two antidepressant medications	CAPS, PCL–5	Recent trauma (most frequent: Combat exposure 53.3% and Sexual assault 33.3%)	Repeated Ketamine Infusions (*concomitant therapy: SRI 13.3%*, *SNRI 33.3%,Tricyclic/heterocyclic antidepressant 13.3%;* *Other antidepressant 46.7%; Mood stabilizer 33.3%* *APS 13.3%; BDZ 13.3%; Z-drug sedative-hypnotic 33.3%; Stimulant 20%;* *Opiate 20%; Prazosin 26.7%*	Repeated ketamine infusions in a comorbid population was associated to rapid and sustained improvement in PTSD and depression symptoms
Chamberlain et al.^59^	2019	UK	Good	Observational study	4 groups of participants: 1. TRD, 2. treatment-responsive depression, 3. untreated depression 4. healthy volunteers.	102^a^	*Not available*	36.5	*Not available*	SCID, HAM-D, ATRQ, BDI	At least one adequate trial with monoaminergic drug *(during study: SSRI 70% , SNRI 15%, mixed reuptake inhibitors 25%, tricyclic antidepressants 4%, mood stabilizers 4% and dopamine receptor antagonists 3%)*	CTQ	Early trauma	-	Increased CRP and treatment-resistance were associated with aspects of clinical heterogeneity in depression including a history of childhood adversity
O’Brien et al.^42^	2019	USA	Good	Observational study	TRD patients with moderate to very severe depressive symptoms (QIDS-SR > of 10)	115	81 (70,43%)^b^	43.78	CTQ-ml 1: 37.63 CTQ-ml 2: 44.46 CTQ-ml 3: 53.13 CT-ml 4: 49.50 CTQ-ml 5: 46.60	QIDS-SR	At least one trial of antidepressant medication	CTQ	Early trauma	Intravenous Ketamine (single infusion or repeated infusion)	Ketamine could be more effective in TRD patients with more childhood trauma burden
Minelli et al.^57^	2019	Italy	Good	Single-blind randomized controlled trial	TRD patients with at least three documentable traumatic events over their lifetime	22		TF-CBT group: 53.3 EMDR group: 52.3		SCID-I, MCMI-III, MADRS BDI-II	At least 2 adequate trials of 2 different classes of antidepressants and to an adequate trial of a TCA	CECA-Q, Paykel Scale of stressfull life events, HRLSI	Both early and recent trauma	Trauma-focused therapy (EMRD an TF-CBT)	Evidence-based trauma-focused psychotherapies, particularly EMDR, can represent effective interventions to treat TRD patients
Nikkheslat et al.^60^	2020	UK	Good	Cross-sectional study	4 groups of participants: 1. TRD, 2. treatment-responsive depression, 3. untreated depression 4. healthy volunteers	80^a^	*Not available*	36.8	*Not available*	SCID, HAM-D, BDI-II, ATRQ	At least 6 wks of treatment with one or more monoaminergic antidepressants	CTQ	Early trauma	-	Treatment non-responder patients had higher exposure to childhood trauma than responders. The severity of childhood trauma was associated with increased diurnal cortisol levels in individuals with glucocorticoid resistance
Wilkes et al.^61^	2020	USA	Good	Retrospective chart review	rTMS patients at Tripler Army medical center	77	29 (37.66%)^c^	39.5	Not	BDI	At least three different antidepressant medication most common: 91% SSRI; 63.6% NDRI; 51.9% SNRI	PCL	Recent trauma	rTMS	rTMS treatments may produce a reduction in symptoms of both depression and PTSD in patients with refractory depression and comorbid PTSD
Yrondi et al.^34^	2020	France	Good	Prospective cohort study	French cohort of outpatients with TRD^d^	256	*Not available*	53.21	*Not available*	MINI; MADRS, QIDS‐SR	At least two adequate trials of different classes of antidepressants	CTQ	Early trauma	-	Significant association between the severity of depressive disorders and childhood maltreatment (especially physical and sexual abuse and physical neglect) in the TRD population
Yrondi et al.^35^	2021	France	Good	Prospective cohort study	French cohort of outpatients with TRD^d^	256	*Not available*	53.21	*Not available*	MINI; MADRS; ATHF	At least 2 adequate trials of different classes of antidepressants	CTQ	Early trauma	-	Strong association between suicidal behavior and childhood maltreatment (in particular childhood physical neglect) in a TRD population.
Yrondi et al. (B)^36^	2021	France	Good	Prospective cohort study	Geriatric patients from a French cohort of outpatients with TRD^d^	96	*Not available*	67.25	*Not available*	MINI; MADRS, QIDS‐SR	At least 2 adequate trials of different classes of antidepressants	CTQ	Early trauma	-	Association between childhood trauma (mainly relating to PA) and the intensity of depressive symptoms.
McCarthy et al.^62^	2021	USA	Good	Randomized prospective single-blind study	veterans with TRD	182	46 (27%)^c^	TAU group: 50.3 PGX group: 52.5	*Not available*	QIDS-SR CGI	at least 1 adequate trial of an antidepressant or mood stabilizer	Patient interview and historical chart review	Recent trauma	PGX-guided treatment	Only in the PTSD group the PGX test showed a statistically significant benefit
Magalhães et al.^63^	2021	Brazil	Good	Observational study	TRD patients	67	24 (35.82%)	40	*Not available*	MINI-plus 5.0 MADRS	at least two adequate medication trials	ETISR-SF	Early trauma (childhood sexual abuse)	Subcutaneous esketamine	The patient sample had a relatively high prevalence rate of CSA. CSA does not appear to predict poor response to esketamine
Artin et al.^47^	2022	USA	Fair	Open-label retrospective study	Veterans with TRD and PTSD	35		45.4		PHQ–9, MGHATRQ	At least 2 antidepressants (*ECT 23%; rTMS 14%)*	PCL–5	Recent trauma	repeated intranasal (S)-ketamine treatment	Both depression and PTSD symptoms improve with repeated intranasal (S)-ketamine treatment
Rothärmel et al.^64^	2022	France	Good	Open-label, single arm, retrospective pilot study	Patients with TRD and PTSD	11		47.27		MADRS PHQ–9 GAF CGI-SS	At least two adequate trials of two antidepressant	PCL–5	Early or recent trauma *(2 rape, 4 sexual abuse in childhood, 1* *suicide of family member, 1 brutal* *love breakup, and 3 workplace bullying)*	IN Esketamine sessions. *Concomitant medication prescriptions SSRIs* *(n = 2), SNRI* 7, α2 *antagonists (n = 1), tricyclics (n = 1), SGA* *(n = 9), mood stabilizers (n = 10), and BDZ (n = 6)*	Esketamine significantly improved depression symptoms
Bentley et al^65^	2022	USA	Good	Retrospective study	Veterans with TRD and PTSD	15		49.1		PHQ–9	Mean number of antidepressant trials was 2.7 *(ECT 53%* *rTMS 47%* *History of Ketamine 53%)*	PCL–5	Recent trauma	Both IN-(S)-ketamine and IV-(R,S)- ketamine)	Both depression and PTSD symptoms improve with IV-(R,S)- ketamine)
Giampetruzzi et al.^27^	2023	USA	Good	Observational study	TRD patients	454	277 (61.0 %)^e^	49.60	*Not available*	BDI-II; MSM	65.6 % reported at least 3 failures of adequate trial of medications	CTES	Early childhood trauma		Differences in severity symptomatology and treatment outcomes, between patients reporting no ACEs versus 3+ ACEs. Violence and illness/injury were significant predictors of more severe symptomatology. Sexual trauma and violence uniquely predicted a lifetime suicide attempt(s) Sexual trauma predicted lifetime inpatient admission(s)
Hickson et al.^66^	2024	USA	Good	Retrospective cohort study	Veterans	99	51 (51.51%)^c^	*48.14*	*Not available*	PHQ–9 BSS	Failure of more than one antidepressant in the treatment of a current, active episode of an MDD diagnosis	PCL–5	Both early and recent trauma	30 sessions of dTMS treatment using the Hesed coil (H1 coil)	Positive impact of dTMS on symptoms of MDD, PTSD and suicidal ideation among veterans with TRD

^***^Abbreviations used in Table 1 in alphabetical order.

Abbreviations: APS, antipsychotics; ACEs, adverse childhood experiences; APS, antipsychotic; ATHF, antidepressant treatment history form; ATRQ, antidepressant treatment response questionnaire; BDI, beck depression inventory; BDI-II, beck depression inventory-II; BDZ, benzodiazepines; BSS, beck suicide ideation; CAPS, clinician administered PTSD scale; CBT, cognitive behavioral therapy; CECA (CECA-Q), childhood experience of care and abuse questionnaire; CGI-SS, clinical global impression-suicide scale; CM, conversational model; CSA, child sexual abuse; CTES, childhood traumatic events scale; CTQ, the childhood trauma questionnaire; CTQ-ml, CTQ maltreatment load; ECT, electroconvulsive therapy; EMDR, eye movement desensitization and reprocessing; ETI, early trauma inventory; ETISR-SF, early trauma inventory self- reported short form; GAF, global assessment of functioning; HAM-D, hamilton rating scale for depression; HRLSI, holmes-rahe life stress inventory; MADRS, montgomery-asberg depression rating scale; MCMI-III, millon clinical multiaxial inventory; MGHATRQ, massachusetts general hospital antidepressant treatment response questionnaire; MINI, mini-international neuropsychiatric interview; MINI-plus 5.0, mini-international neuropsychiatric interview (MINI)-plus 5.0; MSM, maudsley staging method; NDRI, norepinephrine–dopamine reuptake inhibitor; PA, physical abuse; PCL, PTSD checklist; PCL-5, PTSD checklist for DSM-5; PGX, pharmacogenetic tests; PHQ-9, Patient Health Questionnaire-9; PTSD, post traumatic stress disorder; QIDS-SR, quick inventory of depressive symptomatology; rTMS, repetitive transcranial magnetic stimulation; SCID, structured clinical interview for DSM; SGA , second-generation antipsychotics ; SNRI, serotonin–norepinephrine reuptake inhibitors; SSRI, selective serotonin reuptake inhibitor; PGX, pharmacogenetic tests; TAU, treatment as usual; TCA, tricyclic antidepressants; TF-CBT, trauma focused cognitive-behavioral therapy; TRD, treatment resistant depression.

a Considering group with TRD.

b CTQ maltreatment load at least 1.

c Patients with PTSD comorbidity.

d Same sample.

e At least 1 adverse childhood experiences.

## 3. Results

The search yielded a total of 22 publications, including 18 clinical studies, 3 case reports, and 1 case series, all from the period 2014 to 2024. Details of the individual studies included in the review are listed in Tables 1 and 2.

**Table 2.** Case Report and Case Series Included

Study	Year	Country	Quality rating	Type of study	Sex	Mean age	TRD		Trauma		Type of intervention	Outcome
							Assessment	Previous treatment	Assessment	Type of trauma		
Nakama et al.^67^	2014	Hawaii	Fair	Case report of patient with TRD and PTSD	M	24	Criteria of DSM-IV-TR; BDI	CBT, Prolonged expose to therapy, Sertralina 200 mg/die; Venlafaxine XR 225 mg/die; gabapentin 300 mg/die; quetiapine 300/die; desyrel 300/die; prazosin 5 mg/die; hydroxyzine up to 200 mg/die; zolpidem 10 mg/die, aripiprazole 5 mg/die	Criteria of DSM-IV-TR for PTSD; PCL-M	Recent trauma (repeated war trauma)	L DLPFC rTMS	alleviation of suicidal ideations and most of the PTSD and MDD symptoms after L DLPFC rTMS treatments
Guo et al.^68^	2022	China	Good	Case report of patient with TRD and PTSD	F	16	Criteria of DSM–5; HAMD	Sertraline 100 mg/die; Venlafaxine XR 225 mg/die; Buproprion XR 300 mg/die Diazepam 10 mg/die; lorazepam 0.5 qn; Olanzapina 10 mg/die; MECT	CAPS	Early and recent trauma (sexual assault at 8 ys old; suicide of friends at 15 ys old)	augmentation strategy with Prazosin 1 mg/die	low doses of prazosin (0.25 to 1 mg daily), which gave marked improvements in both PTSD and depression symptoms
Willms et al.^69^	2022	USA	Good	Case report of patient with TRD, PTSD and GAD	M	30	SCID-I C-SSRS PHQ–9	Sertraline 100 mg/die, Vortioxetina 10 mg/die, Buproprion 300 mg/die, Duloxetine 60 mg/die, Amitriptyline 25 mg/die, Clonazepam 0.5 mg/die, Zolpidem 5 mg/die	CAPS–5	Early ad recent (traumatic events associated with his medical training, history of household instability and parental fighting throughout his childhood	8-mo regimen of IV ketamine infusions and, two KAP sessions, and two psychotherapy sessions	Positive effects of ketamine both immediate and long-term
Veraart et al.^70^	2023	the Netherlands	Good	Case series of patients with TRD and PTSD	F	65	HAM-D IDS-SR	SSRIs and SNRIs, mirta- zapine, bupropion, TCAs, lithium, (MAOIs), pregabaline, quetiapine, doxazosine and benzodiazepines; EMDR	PCL–5	Early trauma (maltreatment and emotional neglect in her early childhood and repeated sexual abuse)	oral esketamine plus psychological treatment	Broader “window of tolerance” during exposure and improvement of symptoms
					F	66		SSRIs, mirtazapine, lithium augmentation, and a single dose of psilocybin (dose unknown) through an experimental trial. EMDR		Early trauma (affective neglect and repeated sexual abuse.)		Depressive symptoms as assessed by the HDRS decreased whereas the IDS-SR increased from 35 to 45. EMDR was re-initiated with positive results
					F	57		citalopram, venlafaxine, nortriptyline, lithium augmentation, and tranylcypromine. CBT interpersonal therapy)		Recent trauma (she had witnessed a deadly attack)		during her stay someone was verbally aggressive with her. After this event, there was increase of anxiety and suicidal ideation. the treatment was discontinued due to absence of mood improvement
					F	34		SNRIs, TCAs, lithium, pregabalin, mirtazapine, APS, and MAOIs, ECT, and schema therapy		Early trauma (physical, mental, and sexual abuse in her Youth)		improvement on the tolerability of emotions concomitant with the re-experiencing of memories of the traumatic event
					F	54		SSRIs, TCAs, lithium, bupropion, MAOI, and several BDZ, ECT, EMDR		multiple traumas in the past, including sexual abuse and isolation on a psychiatric ward.		Improvement of PTSD and depressive symptoms

*Note:* Abbreviations used in Table 2 in alphabetical order.

Abbreviations: APS, Antipsychotics; BDI, Beck Depression Inventory; BDZ, Benzodiazepines; C-SSRS, Columbia Suicide Severity Rating Scale; CAPS-5, Clinician-Administered PTSD Scale for DSM-5; CAPS, Clinician Administered PTSD Scale; CBT, Cognitive behavioral therapy; DSM-5, Diagnostic and Statistical Manual of Mental Disorders Fifth Edition; DSM-IV-TR, Diagnostic and Statistical Manual of Mental Disorders Text Revision; ECT, Electroconvulsive therapy; EMDR, Eye Movement Desensitization and Reprocessing; GAD, Generalized Anxiety Disorder; HAM-D, Hamilton Rating Scale for Depression; IDS-SR, The Inventory of Depressive Symptomatology; KAP, ketamine-assisted psychotherapy; L DLPFC rTMS, left dorsolateral pre- frontal cortex Repetitive Transcranial Magnetic Stimulation; MAOI(s), Monoamine oxidase inhibitors; MDD, Major depressive disorder; MECT, modified electroconvulsive therapy; PCL-5, PTSD Checklist for DSM-5; PCL-M, PTSD CheckList - Military Version; PHQ-9, Patient Health Questionnaire-9; PTSD, Post Traumatic Stress Disorder; rTMS, Repetitive Transcranial Magnetic Stimulation; SCID-I, Structured Clinical Interview for DSM Axis I Disorders; SNRI(s), Serotonin–norepinephrine reuptake inhibitors; SSRI, Selective Serotonin Reuptake Inhibitor; TCA(s), Tricyclic antidepressants; TRD, Treatment Resistant Depression.

### 3.1. Clinical study

#### 3.1.1. Characteristics of the study samples

The research includes 18 clinical studies for a total sample of 1711 patients with TRD, with a mean age of 46.92 years. Considering the studies for which data are available, 735 patients (42.96%) were exposed to at least 1 traumatic event, of which 202 reported a diagnosis of PTSD. In this calculation of the TRD sample, the “untreated depression,” “depression with response to treatment,” and “healthy controls” groups, which were present in the total sample of 2 studies, were excluded, and the FACE-DR cohort of the 3 studies by Yrondi et al. was included once.^34^ ^-^^36^ In most studies (n=10, 55.55%), TRD was investigated in association with early trauma; in 5 studies (27.78%), participants reported recent trauma; and, in 3 studies (16.67%), both early and recent trauma.

#### 3.1.2. Assessment

Most studies used several scales to assess MD, 3 studies (16.67%) used only 1 scale. The Structured Clinical Interview for DSM Disorders (SCID) was used in 5 studies (27.78%) as well as the Mini-International Neuropsychiatric Interview (MINI) in 5 studies (27.78%). The most frequently used scales to assess MD were: in 7 studies the Montgomery-Asberg Depression Rating Scale (MADRS) (38.89%); in 4 (22.22%) the Hamilton Rating Scale for Depression (HAM-D); in 4 (22.22%) the Patient Health Questionnaire (PHQ-9); in 4 (22.22%) the Quick Inventory of Depressive Symptomatology - Self Report (QIDS-SR); in 4 studies (22.22%) the Beck Depression Inventory (BDI); in 3 studies (16.67%) the BDI-II. The Global Assessment of Functioning (GAF) and the Clinical Global Impression Scale (CGI) were used twice, the Beck Suicide Ideation Scale (BSS) was used once.

Regarding the definition of TRD:

- 8 studies (44.44%) reported cases of patients with at least 2 previous adequate trials of antidepressants, of which 5 studies stated that these antidepressants belonged to different classes, and 1 article did not specify whether the drugs used were antidepressants but mentioned “medications,”
- 2 studies (11.11%) included patients with at least 3 trials of antidepressants as well as other drug classes (BDZ; mood stabilizers), ECT, or psychotherapy,
- 5 studies (27.78%) reported at least 1 adequate trial of antidepressants,
- 1 study (5.55%) included patients with at least 1 adequate trial of antidepressants or mood stabilizers, and
- 1 study reported the average number of antidepressant trials (2.7), while another study reported that 65.6% of the sample had at least 3 failed trials of antidepressants, with no information available for the remaining 33%.

Only 6 studies (33.33%) adopted specific questionnaires to assess TRD, namely, the Antidepressant Treatment History Form (ATHF) in 2 cases, the Antidepressant Treatment Response Questionnaire (ATRQ) in 2 cases, the Maudsley Staging Method (MSM) in 1 case, and the MGHATRQ in 1 case.

The most frequently used scale to assess the presence of trauma was the Childhood Trauma Questionnaire (CTQ) (n = 7; 38.89%). 5 studies (27.78%) used the Post-traumatic Stress Disorder Checklist 5 (PCL-5), and 1 study (5.55%) used the Post-traumatic Stress Disorder Checklist (PCL). The Childhood Experience of Care and Abuse Questionnaire (CECA) was used twice, while the Clinician-Administered PTSD Scale (CAPS), the Early Trauma Inventory (ETI), the Early Trauma Inventory Self-Reported Short Form (ETISR-SF), the Childhood Traumatic Events Scale (CTES), the Paykel Scale of stressfull life events, and the Holmes-Rahe Life Stress Inventory (HRLSI) were only used once.

#### 3.1.3. Clinical features of TRD

Eight studies focused on the clinical features of TRD (ie, suicidal ideation, course, or biochemical parameters), emphasizing an association between treatment response and childhood trauma. Childhood adversity was not only common in TRD patients but also associated with poor clinical course (1 study), psychosis (1 study), suicidal behavior (3 studies) and, especially, sexual trauma, with lifetime inpatient admission(s) (1 study). Three studies found that childhood maltreatment was significantly related to the severity of depressive symptoms in TRD patients. According to one of them, this association is evident, especially for physical and sexual abuse and for physical neglect. In another study, the severity of childhood trauma was associated with increased diurnal cortisol levels in individuals with glucocorticoid resistance.

#### 3.1.4. TRD treatments

Eleven studies focus on possible TRD treatment:

- 2 studies highlighted a positive response to trauma-focused psychotherapy,
- 6 studies investigated response to pharmacotherapy (esketamine or ketamine): 4 emphasized improvements in both depressive and post-traumatic symptoms; 1, a stronger response in subjects with greater burden on the CTQ; and in 1 study, childhood sexual abuse (CSA) does not seem to predict poor response to esketamine,
- 2 studies showed a good response, both for depressive and trauma-related symptoms, to repetitive transcranial magnetic stimulation (rTMS) in patients with refractory depression and comorbid PTSD, and
- 1 study suggests the benefit of pharmacogenomic (PGX) testing in patients with TRD and PTSD. This benefit was not found in patients with comorbid bipolar disorders or MDD.

### 3.2. Case series and case report

#### 3.2.1. Characteristics of the study samples

This research comprised 3 case reports and a case series with 5 case descriptions, with a total of 8 patients. The patients described were predominantly women (6 cases, 75%) with a mean age of 43.25 years. All reported cases showed comorbidities between TRD and PTSD. In 1 case, there is also comorbidity with generalized anxiety disorder. Three patients reported an early trauma, two reported recent trauma, two reported both early and recent multiple trauma, and one case reported undated multiple trauma.

#### 3.2.2. Assessment instruments

The diagnosis of MD was made in 2 cases according to the DSM criteria and in 1 case using the SCID. In addition, the assessment of depressive symptoms was made in 6 cases with the HAM-D, in 5 cases with the Inventory of Depressive Symptomatology-Self report (IDS-SR), in 1 case with the PHQ-9, in 1 case with the BDI, and in 1 case with the Columbia-Suicide Severity Rating Scale (C-SSRS). All patients had undergone adequate trials of at least 2 antidepressants, so they were treatment-resistant according to the definition most used in the literature. PTSD was assessed in 3 cases by CAPS and in 5 cases by PCL-5.

#### 3.2.3. Type of intervention and outcome

In 5 cases, the patients received oral esketamine plus psychological treatment, which led to positive results in 4 cases. In 1 of the cases, a traumatic event (verbal aggression towards the patient) during hospitalization was reported with a worsening of the clinical picture.

In the other 3 cases, all of which showed a positive result, a left dorsolateral prefrontal cortex (L-DLPFC) rTMS, an augmentation strategy with prazosin or IV ketamine infusions with ketamine-assisted psychotherapy (KAP) sessions, and 2 psychotherapy sessions were used.

## 4. Discussion

The purpose of the present review was to summarize the current state of knowledge on the relationship between trauma and TRD to improve understanding of the role of trauma in TRD. Despite an apparent consensus on the definition of TRD, a first emerging issue is the heterogeneity in TRD assessment across studies.^37^ ^,^^38^ The globally accepted definition of TRD is based on an inadequate response to consecutive treatment with 2 compounds with different mechanisms of action (eg, an SSRI and an SNRI) taken over a sufficient period of time at an adequate dose.^5^ ^,^^37^ This definition, which is also used by the EMA, is based on 2 concepts: I) inadequate response to 2 drugs of different pharmacological classes is more difficult to treat than inadequate response to 2 drugs with the same mechanism of action (eg, 2 SSRIs); II) switching treatment within a class is less effective than switching to a different pharmacological class. However, the EMA itself emphasizes in the “Guidelines on clinical investigation of medicines for the treatment of depression” that these assumptions are not confirmed by the literature and therefore the TRD guidelines must be considered when there is a lack of response to appropriate treatment with at least 2 different antidepressants of different or the same class.^5^ Other definitions of TRD also emerged, such as those assuming “failed” trials from 1 or more classes of pharmacological treatments, including non-pharmacological treatments (such as brain stimulation or ECT), and may vary with the same definition of “treatment failure.”^38^ ^,^^39^ ^,^^40^

In most of the studies included in this review, TRD was diagnosed in the presence of MD (unipolar or bipolar) that did not respond to at least 2 adequate trials of antidepressants. However, some studies did not specify whether the antidepressants were of the same or a different class, and other studies included other drug classes, ECT, or psychotherapy in different trials. Furthermore, 6 studies included patients who had not responded to at least 1 adequate trial, and only 6 studies used specific assessment tools and models.

The dishomogeneity in the methodology of the included studies was also evident in the aspects related to the assessment of trauma and its role in TRD, the core of this review. This heterogeneity explains the variability in the prevalence of trauma across the studies examined, ranging between 27% and 100%, without taking into account the 8 case reports including patients with TRD and PTSD, as well as the studies in which the presence of trauma is an inclusion criterion. It should also be considered that, in some studies, the prevalence of patients with a traumatic event is not available as the trauma/TRD association was assessed as an association between assessment tool scores (ie, CTQ).

In the literature, several studies on depression emphasize the role of childhood trauma,^2^ ^,^^27^ ^,^^30^ which is indeed the most researched, even when the studies focus on TRD. However, in 29.41% of the included studies, patients had recent trauma, and in 11.76%, both early and recent trauma.

An important finding of the present study is the fact that despite patients with recent trauma showing comorbidities between TRD and overt PTSD, a proportion of patients with early trauma had no overt trauma-related symptomatology. Consistently, Stevenson et al. ^41^ pointed out that, in their sample, the trauma had not emerged at the first interview but only during the full assessment. The presence of a traumatic event is not only common but also appears to influence clinical (ie, severity of symptoms, suicidal ideation, and psychosis), biochemical (elevated diurnal cortisol levels in individuals with glucocorticoid resistance and elevated C-Reactive Protein), and treatment-related aspects, according to the results of the studies examined. In this regard, we may argue the fact that among the papers investigating ketamine or esketamine pharmacological treatment of TRD reported improvement not only in depressive but also, when present, in post-traumatic symptoms. O’Brien et al.^42^ also concluded that ketamine may be more effective in TRD patients with higher childhood trauma burden. One explanation for this phenomenon could be the effect of ketamine on trauma-related behavioral sensitization processes by attenuating the hyperexcitation and depressive symptoms that are their expression.^42^ ^-^^44^ Ketamine has also been shown to accelerate the extinction and reconsolidation of fear.^45^ ^,^^46^ Artin et al.^47^ reported a perceived decoupling of emotion-cognition with ketamine in the veterans involved in the study, which could interrupt maladaptive patterns of rumination and avoidance. Intravenous ketamine (KET-IV) was found to be safe and well-tolerated in the improvement of depressive, anxiety, and functionally impaired symptoms of adults with TRD.^48^ The results in the literature confirm the efficacy of ketamine in both the KET-IV and intranasal esketamine (ESK-NS) forms in TRD subjects, including those with suicidal ideation. KET-IV shows a significantly greater antidepressant effect than ESK-NS during short-term follow-up periods.^49^ Although not used in any of the included studies, we recall for the sake of completeness that there is a growing interest in the use of other psychedelics besides ketamine, both atypical (3,4-methylenedioxymethamphetamine-MDMA) and classical (lysergic acid diethylamide-LSD, psylocybin, …) in TRD and PTSD. For both disorders, the evidence for efficacy is very limited, but the studies seem promising.^49^ ^-^^52^

Among the included articles, 2 studies and 1 case report treated patients with TMS with good results. Increasing evidence supports the efficacy of neuromodulation techniques such as rTMS and iTBS (theta burst stimulation) in TRD^53^ ^,^^54^ and highlights their comparable efficacy with other approved treatments, such as ESK-NS.^55^ Additionally, from a neurobiological perspective, rTMS appears to act on the executive control network and may modulate hyperactive networks (eg, the Default Mode Network). Consistently, it acts on the “top-down” areas of the PFC that regulate Brodmann Area 25 (BA25), which is hyperactive in depression; increased BA25 activity has also been closely associated with stress-related disorder of depression, and stress is thought to be the trigger for BA2 activation.^56^

An improvement in depressive symptoms was also reported in the 2 studies on psychotherapeutic interventions in patients with TRD; in both cases, trauma-focused psychotherapies, such as Eye Movement Desensitization and Reprocessing (EMDR), Trauma-Focused Cognitive Behavioral Therapy (TF-CBT), or trauma-informed therapy as in the conversational model (CM), along with pharmacotherapy, were used.^57^ ^,^^41^ The effectiveness of therapies targeting the trauma core seems to confirm that trauma can be considered an important factor in TRD.

When discussing our results, some limitations must be taken into account. First, we only considered articles in English. In order to maintain the quality of the included papers, we excluded preprints or abstracts, with the risk of missing some information. Given the paucity of data in the literature, we also included case reports, but a possible limitation could also be related to the fact that these may be anecdotal and inherently biased. In addition, some of the included studies have a small sample size, which may affect the statistical power of the study itself. Finally, the lack of homogeneity in the definition of TRD goes hand in hand with the clinical diversity in patients with the same diagnosis of depression and with the different aims of the studies in question, which means that heterogeneity is inevitably a limitation of research on TRD, despite the restrictive selection criteria.

## 5. Conclusion

In summary, the present review highlights the role of early and recent trauma in TRD, even in cases where no post-traumatic symptomatology is evident, pointing to the need for a thorough assessment of trauma in patients with TRD and its role as a therapeutic target. However, it also highlights the need to establish a clear definition with standardized assessment methods for TRD in order to make future clinical trials more homogeneous. Acquiring knowledge about TRD can help improve the organization of mental health services, leading to changes in practice in response to the growing clinical interest.

## Data Availability

All data generated or analyzed during this study are included in this published article.
